# Supplementary material for: S100A4 Is a Biomarker of Tumorigenesis, EMT, Invasion, and Colonization of Host Organs in Experimental Malignant Mesothelioma
Source: Cancers (Basel). 2020 Apr 10;12(4):939. doi: 10.3390/cancers12040939 (PMC7226589; doi:10.3390/cancers12040939)
Supplement: Supplementary file 1 [file cancers-12-00939-s001.zip › cancers-754731-supplementary.docx]

Supplementary Material


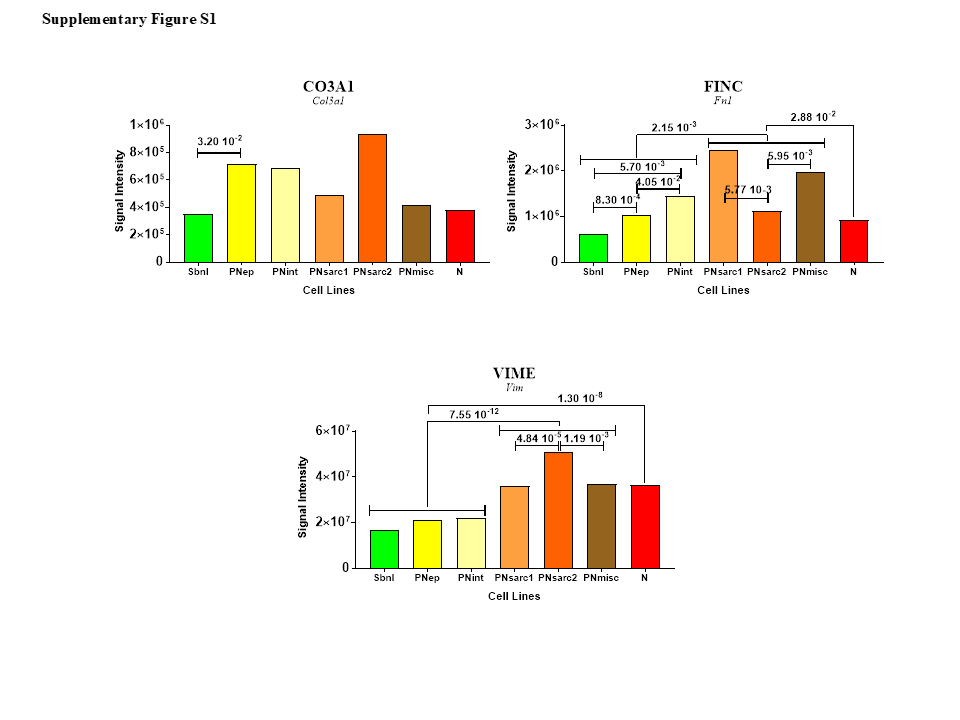


**Figure S1.** Comparative proteomic abundances of EMT (epithelial-to-mesenchymal transition) markers in the different groups and subgroups of rat mesothelial cell lines, described in Table 1. When *p* < 0.05 was observed, values are indicated at the top of the bars. CO3A1, Collagen alpha-1 (III) chain; FINC, Fibronectin; VIME, Vimentin. Corresponding genes are indicated below in italics.


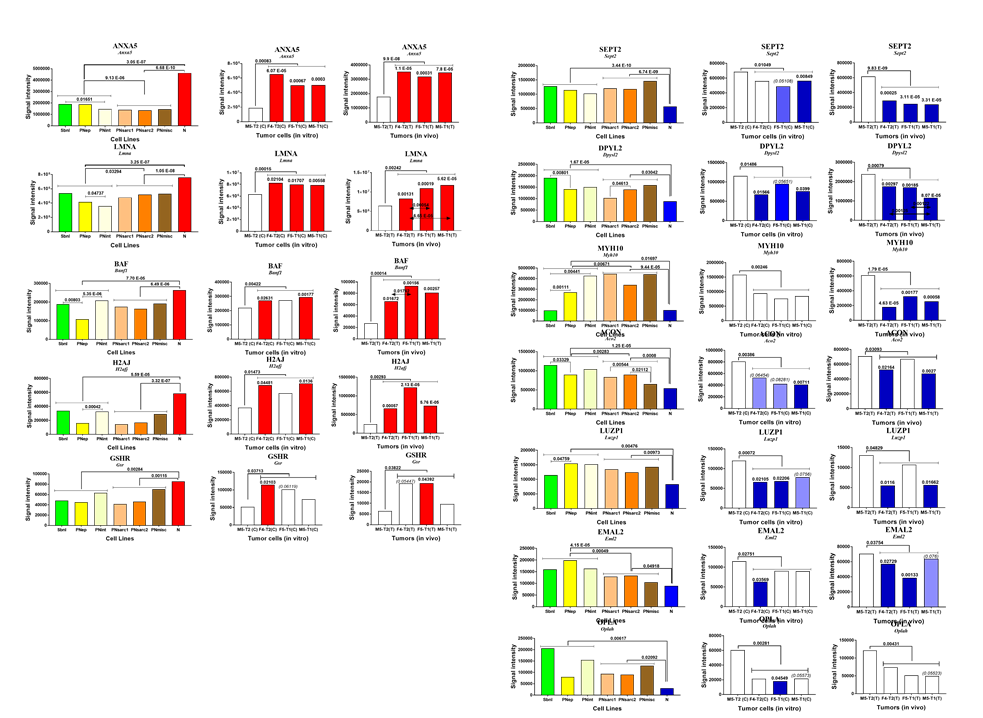


**Figure S2:** Evolution of proteomic abundances for the 12 additional major candidate biomarkers (complement to S100A4 shown in Figure 1(C). Significant increases in red and decreases in blue, with *p* values: Left, comparison between the different subgroups and groups of preneoplastic and neoplastic cell lines (in vitro); middle, comparison between invasive and non-invasive neoplastic cell lines (in vitro); right, comparison between invasive and non-invasive MM (malignant mesothelioma) tumors (in vivo). Blank bars correspond to the absence of significant differences between cell lines/tumors. Strong tendencies are indicated with lighter red/blue colors and *p* values in italics.


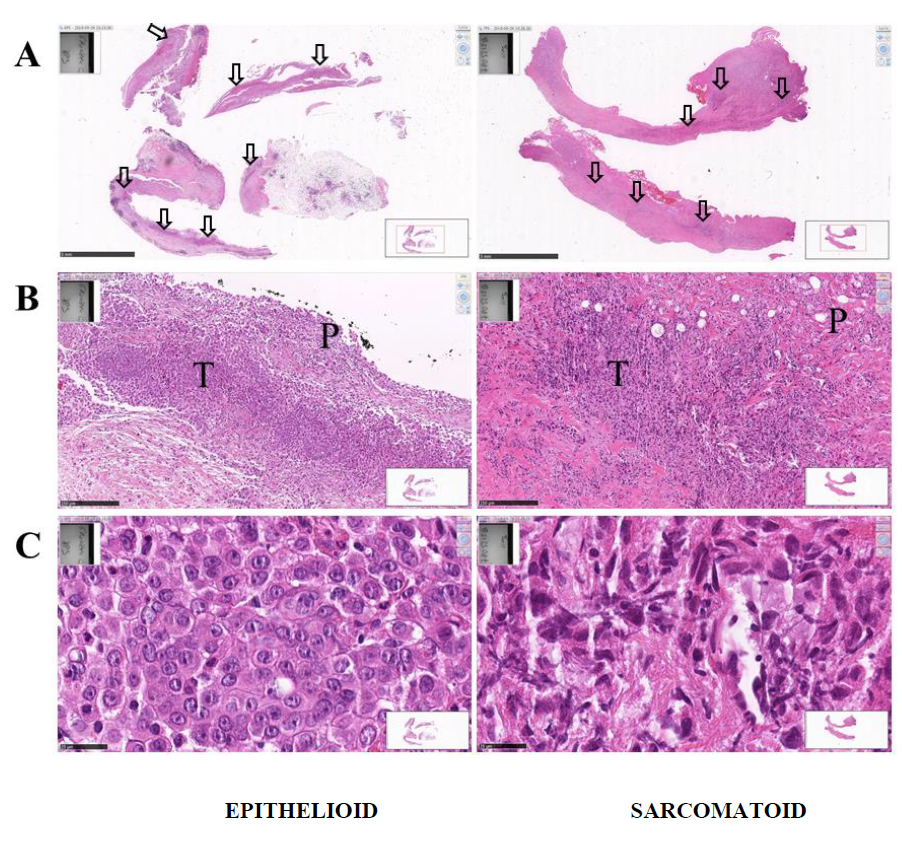


**Figure S3.** Sections of each human MM (malignant mesothelioma) tumor histological subtype, stained with HPS (hematoxylin-phloxine-saffron).The two paraffin-embedded human MM tumor pieces were obtained from samples of the Tumor Bank of the Reims University Hospital Biological Resources, Collection n° AC-2019-340, declared at the Ministry of Health, according to the French Law for the use of tissue samples for research. **A**. General views of tumor samples, scale bars = 5 mm. Areas selected for proteomic analyses are indicated with opened black arrows. **B**. For each area of interest, two different samples were removed with a scalpel from 5 thicker (20 µm) sections, tumor cell clusters (T), and their periphery (P) (×100, scale bars 250 µm). **C.** High magnification views of tumor cell clusters (×800, scale bars 25 µm).


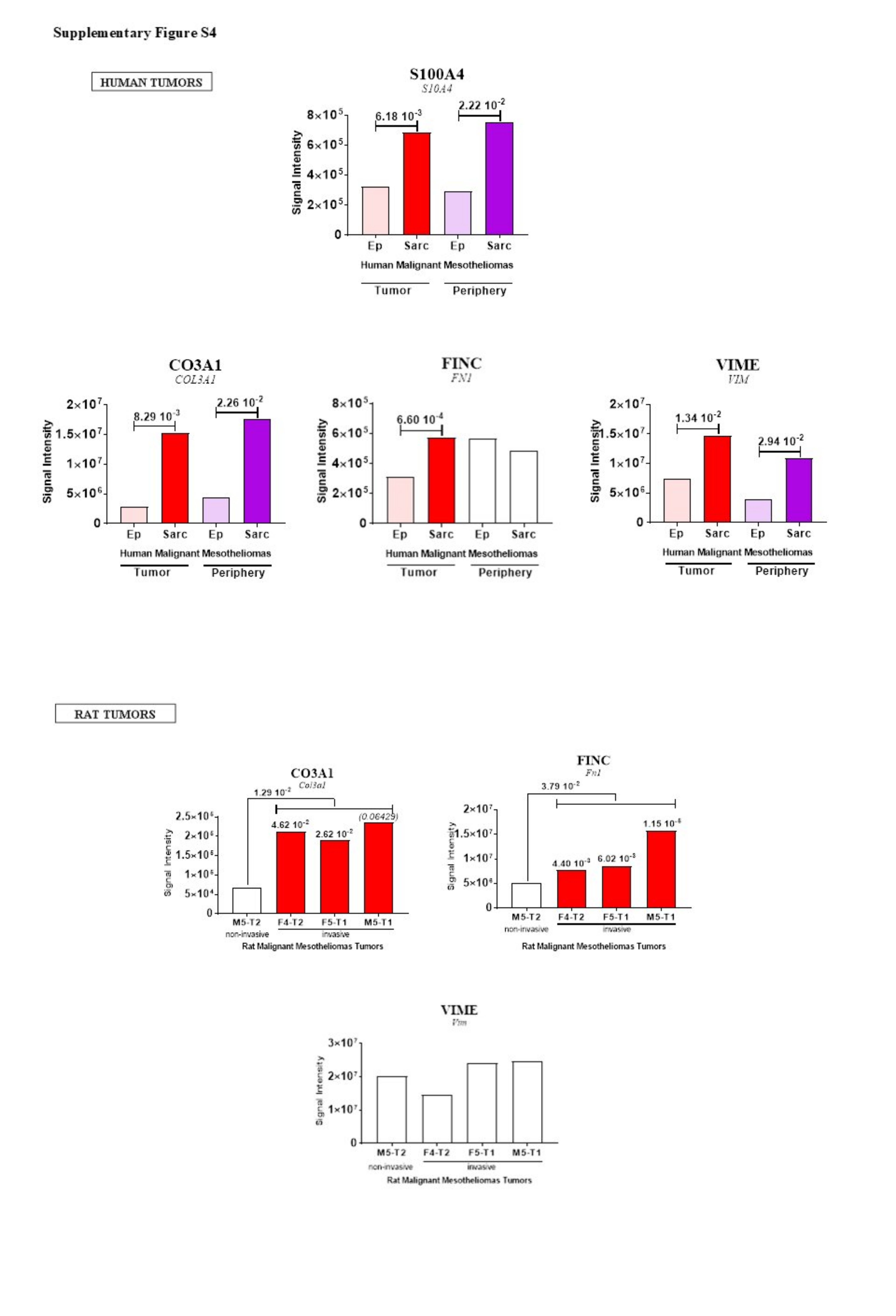


**Figure S4:** Proteomic abundances of protein S-100A4 and EMT markers in human MM tumor samples (data extracted from Supplementary Table S1). Comparison between epithelioid (Ep) and sarcomatoid subtypes for tumor (T) and periphery (P) areas, shown in Supplementary Figure S3. When *p* < 0.05 was observed, values are indicated at the top of the bars. Blank bars correspond to the absence of significant differences. Variations in protein abundances for the same EMT markers in rat MM tumors are provided at the bottom of the figure for comparison, in parallel to protein S-100A4 data illustrated in Figure 1C.


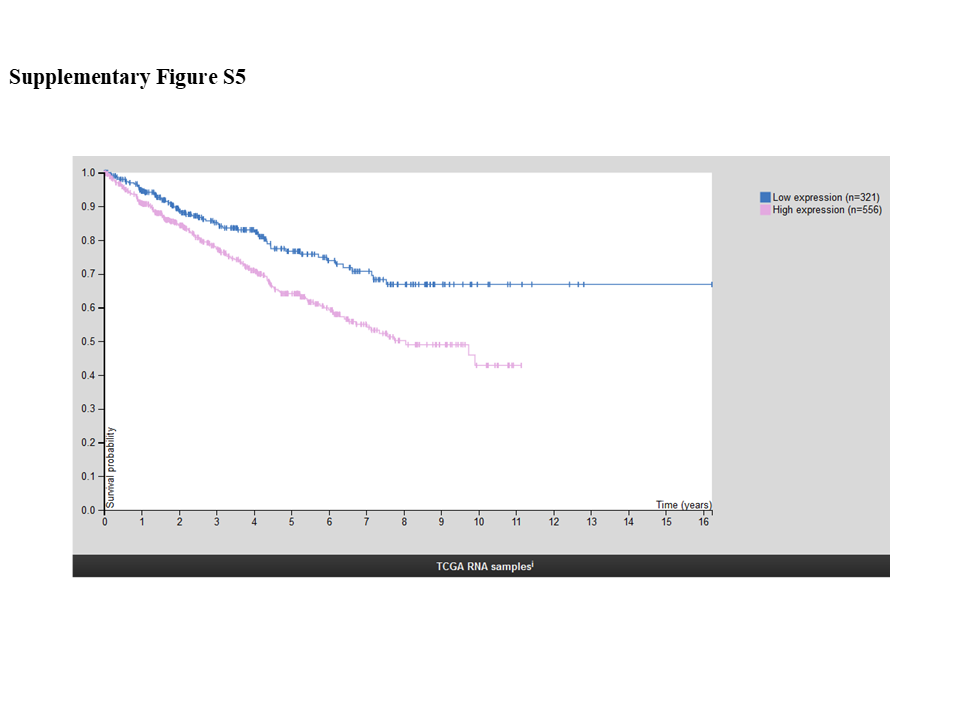


**Figure S5:** Illustration of the impact of protein S-100A4 expression on survival probability for patients with renal cancer. The human protein atlas/Pathology atlas (https://www.proteinatlas.org/ENSG00000196154-S100A4/pathology/renal+cancer).


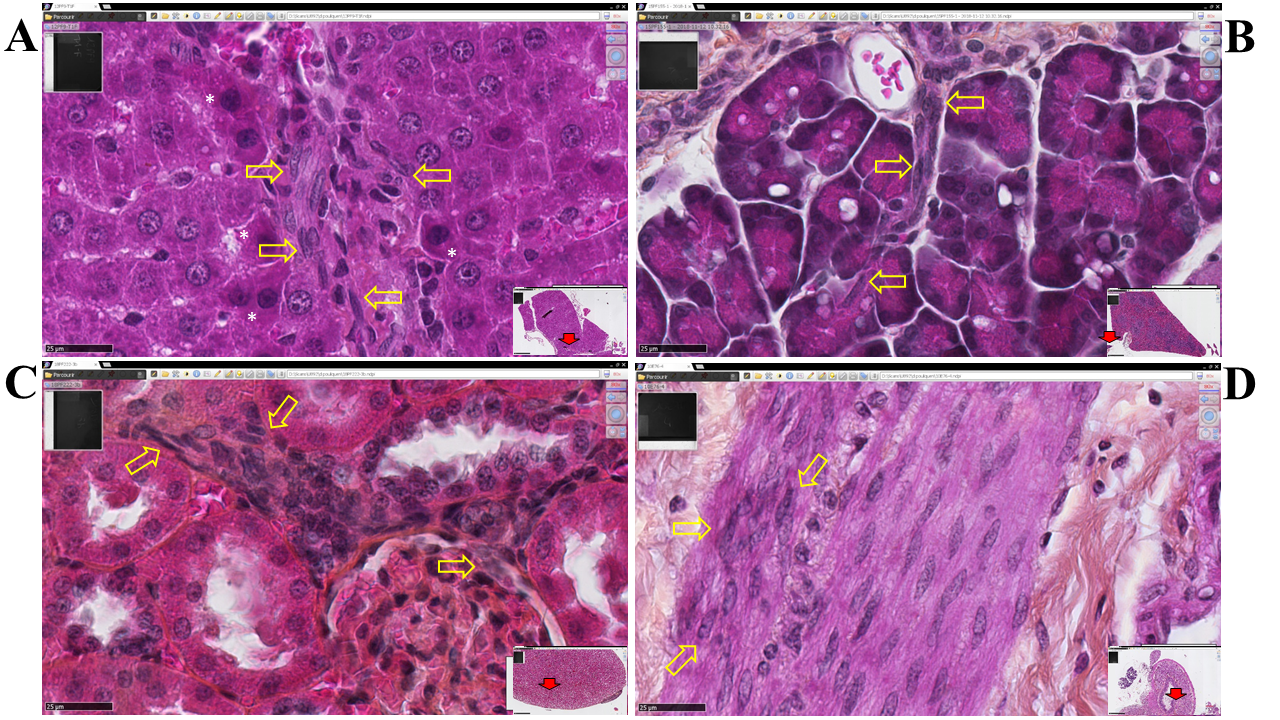


**Figure S6.** M5-T1 rat mesothelioma tumor cells invading secondary organs and tissues. High magnification views (×800, the scale bars represent 25 µm). Inserts show general views (×25), arrows indicating the localization of magnifications, the scale bars represent 1 mm. **(A)**, Liver, asterisks (in white) show pre-apoptotic hepatocytes vicinal to tumor cells exhibiting increased eosinophilia. **(B)**, Pancreas. **(C)**, Kidney. **(D)**, Muscularis externa of the duodenum. The localization of clusters and isolated tumor cells are indicated with yellow arrows.


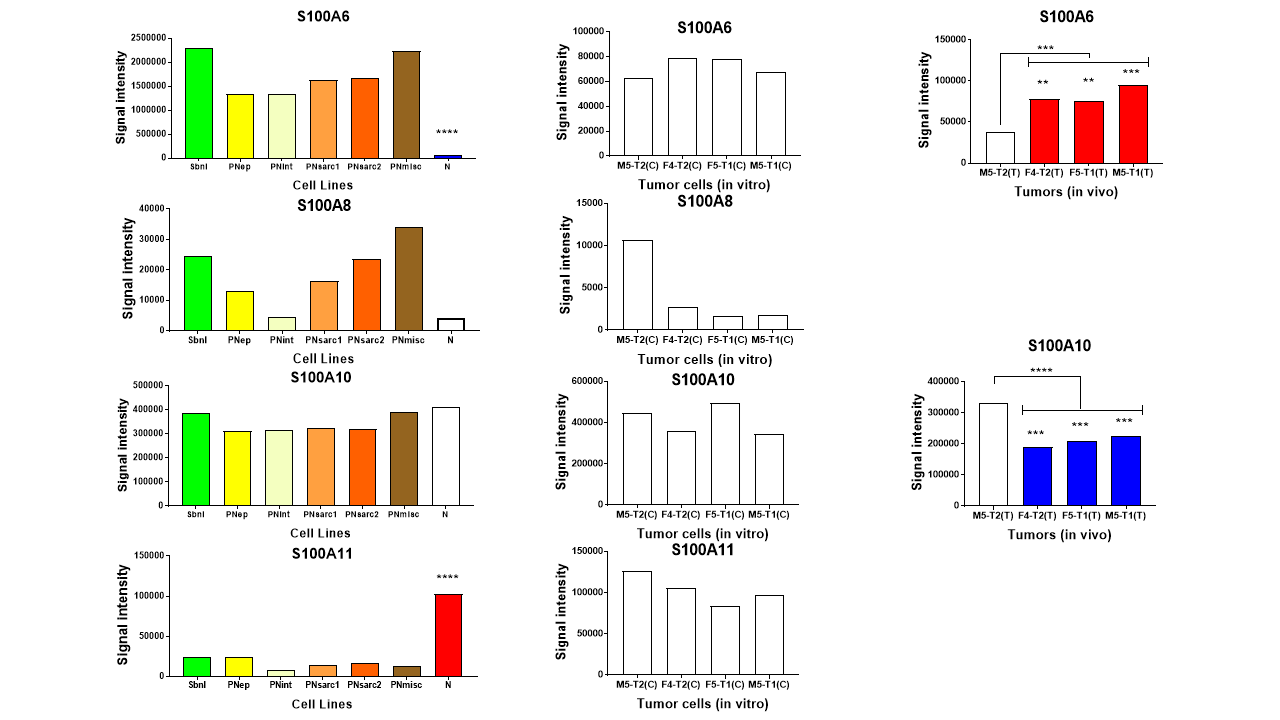


**Figure S7.** Comparative abundances of additional S100 proteins detected in rat mesothelial cell lines and tumors. ** 0.01 < *p* < 0.02, *** 0.001 < *p* < 0.0001, **** *p* < 0.0001. Blank bars correspond to the absence of significant differences between cell lines/tumors.

**Table 1.** Comparative SWATH-MS analysis of human pleural malignant mesotheliomas (sarcomatoid vs epithelioid). Sample preparation followed the same procedure as described in section 4.4 for rat tumors. Spectral library generation, relative quantification, data extraction and statistical analysis were previously described in [68]. The list of data is restricted to all proteins with *p* < 0.06 for clarity. All proteins with *p* < 0.05 are highlighted in yellow. Sections of each tumor subtype, stained with HPS, are illustrated in Supplementary Figure S3. Ep-MM, Sarc-MM, Sarc-Periph, and Ep-Periph correspond to areas selected for proteomic analysis and denoted with “T” (Tumor areas) and “P” (Periphery of the tumors) for each histological subtype (as shown in Supplementary Figure S3-B).

(1): Sarc-MM vs. Ep-MM.

(2): Sarc-Periph vs. Ep-Periph.
